# Shelf Life Prediction of Picric Acid via Model-Based Kinetic Analysis of Its Thermal Decomposition

**DOI:** 10.3390/ma15248899

**Published:** 2022-12-13

**Authors:** Roberto Sanchirico, Marco Luca Santonocito, Valeria Di Sarli, Luciana Lisi

**Affiliations:** 1 Istituto di Scienze e Tecnologie per l’Energia e la Mobilità Sostenibili (STEMS), Consiglio Nazionale delle Ricerche (CNR), via Guglielmo Marconi 4, 80125 Napoli, Italy; 2 Segretariato Generale della Difesa e Direzione Nazionale degli Armamenti, Direzione degli Armamenti Terrestri, Ufficio Tecnico Territoriale Armamenti Terrestri, Piazzale degli Eroi 1, 00048 Nettuno, Italy

**Keywords:** energetic materials, picric acid, aging, shelf life prediction, thermal decomposition, model-based kinetic analysis, differential scanning calorimetry, high performance liquid chromatography

## Abstract

A priori knowledge of the shelf life of energetic materials (EMs) is relevant due to its direct association with safety and functionality. This paper proposes a quick and reliable approach to predicting the shelf life of EMs whose thermal decomposition is an autocatalytic process once their failure threshold has been defined as a function of the limiting extent of conversion. This approach is based on the assumption of a kinetic law consistent with the autocatalytic behavior and on the subsequent extraction, via a suitable procedure of parameter identification, of the kinetics of thermal decomposition from differential scanning calorimetry (DSC) data gathered under dynamic conditions at three different heating rates. Its reliability is proven for picric acid (PA) through the comparison of kinetic predictions with evaluations of conversion obtained by using high performance liquid chromatography (HPLC) analysis for samples subjected to isothermal and non-isothermal accelerated aging tests, as well as for a sample of naturally aged material, i.e., PA, stored at room temperature for more than 10 years.

## 1. Introduction

Energetic materials (EMs) (i.e., explosives, propellants, and pyrotechnics) are thermodynamically unstable substances—if they exist, it is only for kinetic reasons. Most of these materials undergo slow chemical decomposition at room temperature and even more so at elevated temperatures. Thermal stability is strictly dependent on the chemical nature of the substance—aromatic and aliphatic nitro compounds, aliphatic nitramines, and organic azides are considered to be relatively stable, whereas aliphatic nitrate esters are generally less stable [1].

The occurrence of decomposition causes the aging of EMs with the consequent compromise of storage and handling safety as well of functionality. For example, if EMs are exposed to higher temperatures during storage, their aging process is accelerated, and the resulting loss of thermal stability can lead to failure or accidental ignition with potentially catastrophic consequences, such as the explosion that occurred at the port of Beirut in 2020. Therefore, it is essential to know the shelf life of EMs in advance, i.e., the time interval during which they can be stored, handled, and used without any danger [2].

Aging studies are relevant regarding assessing the safe and reliable use of EMs. However, at ambient temperature, the degradation process of EMs is usually too slow for aging studies to be conducted in reasonable times; thus, accelerated aging procedures (i.e., artificial aging treatments at higher temperatures) are generally used to reduce the time scale for such studies. The thermal stability of EMs can be investigated using different test methods based on accelerated aging, the most elaborate of which is the method for the prediction of their shelf life [2]. This method involves accelerated aging at different temperatures (typically between 40 °C and 80 °C) for relatively long time periods (months to years) with different aging time intervals, followed by analysis of the aging-induced changes. A subsequent kinetic analysis and Arrhenius evaluation creates effective activation energy for calculating shelf life at standard storage temperature (in other words, the shelf life is obtained via extrapolation to lower temperatures). A practical example is given in Ref. [3] for a nitrocellulose-based propellant whose accelerated aging process was tracked using measurements of the content of the stabilizer performed by using high performance liquid chromatography (HPLC) analysis. Besides suffering from some important limitations, including its exclusive application to isothermal aging (whereas real life storage conditions often include a complex history of temperature variations) [4], this method is extremely time and money consuming [2]. Therefore, in order to predict the shelf life of EMs, more effective approaches are necessary.

Thermal analysis techniques, especially thermogravimetry (TG), differential thermal analysis (DTA), and differential scanning calorimetry (DSC), have been widely used to investigate the decomposition of EMs (sometimes in combination with Fourier-transform infrared (FTIR) spectroscopy or mass spectrometry (MS) for the identification of reaction products) [5,6,7]. The kinetics of the thermal decomposition of EMs can be extracted from the data gathered by using these techniques. Based on a review of about seventy literature works, EL-Sayed [7] presented data in tabulated form and discussed the values of the kinetic parameters (pre-exponential factor and activation energy) of EMs belonging to various classes, highlighting that these values can vary (even significantly) for the same material based on the measurement technique and the kinetic approach used. This variability is an issue when trying to predict the shelf life of EMs based on their kinetics of thermal decomposition once the failure threshold has been defined as a function of the limiting extent of conversion. Such a prognostic approach is certainly interesting, mostly because it saves time and experimental costs compared to the previously described approach based on accelerated aging tests at different temperatures; thus, some research efforts have been focused on it [8,9,10,11]. Specifically, both model-based [8,9,10] and isoconversional [8,10,11] methods have been adopted. In the case of model-based methods, the kinetic law is predetermined based on the thermal behavior of the substance under examination, whereas this is not needed with isoconversional methods. However, the latter, which are sometimes called “model-free” kinetic analyses, are not assumption-free methods. Burnham and Dinh [8] found as much variability in prediction for various isoconversional methods as between isoconversional methods as a group and different plausible explicit models. However, their predictions (for the conversion of unspecified EMs as a function of time at 80 °C) were not validated against experimental data obtained on actually aged samples.

Li and Cheng [9] extracted the kinetics of the thermal decomposition of nitroguanidine (NQ) in the frame of a model-based approach. The reaction of NQ under isothermal conditions at a temperature of 210 °C was simulated for a total exposure time of 30 min, and the simulated mass loss history was basically consistent with the corresponding experimental curve. Isothermal simulations at temperatures between 150 °C and 210 °C were also performed for a total exposure time of 12 h, showing that before 170 °C, the mass loss raised to only 7%, whereas it shifted rapidly to much more after 170 °C. This is consistent with the change in the mechanism of the decomposition of NQ in the 160–170 °C temperature range inferred by Lee and Back [12] based on the comparison of the values of rate constants they obtained from accelerating rate calorimetry (ARC) experiments with literature values obtained by means of techniques other than ARC.

In order to predict the long term stability of dihydroxylammonium-5,5’-bistetrazolyl-1,1’-diolat (TKX-50), Harter et al. [10] chose an isoconversional method that fitted TG data slightly better than a model-based method also investigated. They also applied the same isoconversional method to other four common explosives, including hexahydrotrinitrotriazine (RDX), octahydrotetranitrotetrazine (HMX), hexanitrohexaazaisowurtzitane (CL-20), and pentaerythritoltetranitrate (PETN). Isothermal predictions at temperatures ranging from 0 °C to 100 °C as well as various climatic predictions of different countries were calculated over a time period of 10 years, whereas the experimental validation was limited to measurements of the mass loss performed after 4, 14, and 28 days of storage of the various explosives at 100 °C. The highest value of mass loss reached after 28 days was that of PETN (~1.4%). Although consistency was claimed between kinetic predictions and experimental data, no direct quantitative comparison was presented.

Kim et al. [11] applied an isoconversional method to DSC data to extract the kinetics of the thermal decomposition of 97.5% RDX, 95% HMX, and boron/potassium nitrate (BPN). They set the reaction progress value of 0.01 after 20 years as the criterion for un-decayed performance of EMs and estimated, through isothermal simulations performed at different temperatures, that 97.5% RDX, 95% HMX, and BPN must be stored at temperatures below 149 °C, 110 °C, and 160 °C, respectively, for a 20-year safe storage. For the validation, only BPN was subjected to accelerated aging tests at a single temperature of 71 °C for 8, 16, 24, and 48 weeks. There was agreement between experimental measurements and kinetic predictions that, even after 48 weeks, the remaining fraction of BPN was very close to unity. However, the negligible degradation of the material did not allow to accurately compare the measured and predicted values of this fraction over the entire aging period.

Although some encouraging preliminary results have been obtained, the above discussion points to the need for further efforts to be devoted to the development of prognostic tools for EMs based on their kinetics of thermal decomposition and for thorough experimental validation of such tools against aging data. The fact that reliable methods/models have not been established yet for specific EMs or specific classes of EMs is also due to a substantial lack of experimental data allowing detailed validation.

The present paper fits in this context with a focus on picric acid (PA), which is an explosive belonging to the class of aromatic nitro compounds that includes, among others, trinitrotoluene (TNT). The thermal decomposition of PA was investigated in this study by means of DSC experiments which confirmed its autocatalytic nature [13,14]. A kinetic law consistent with this nature was thus assumed before extracting the kinetics of thermal decomposition. After a preliminary validation against the DSC experiments, kinetic predictions were compared with evaluations of conversion obtained by using HPLC analysis for samples of PA subjected to isothermal and non-isothermal accelerated aging tests performed over wide ranges of temperature and exposure time and for a sample of naturally aged material, i.e., PA stored at room temperature for more than 10 years. The good agreement found between predictions and corresponding experimental data supports the reliability of the kinetics from this study as a prognostic tool for PA and possibly the extension of the model-based approach adopted to the prediction of the shelf life of EMs that, like PA, decompose in an autocatalytic manner.

## 2. Materials and Methods

Picric acid (PA) was purchased from Sigma-Aldrich (St. Louis, MO, USA). For safety reasons, this material was supplied moistened with water. Dry material was prepared by subjecting the moistened material to vacuum drying at room temperature and then stored in a silica gel desiccator for later use.

Differential scanning calorimetry (DSC) analysis was carried out by using a Perkin Elmer DSC 8000 instrument (Shelton, CT, USA) equipped with an Intracooler II cooling system. For each test, a few milligrams (0.5–2.0 mg) of material were loaded into a 30 μL stainless steel pan that had 150 bar maximum pressure and an operating temperature range of −170 to 400 °C. DSC experiments were performed under both dynamic and isothermal conditions in order to estimate and validate the kinetics of the thermal decomposition of PA (Table 1). At each condition, tests were carried out in triplicate and mean curves were considered.

**Figure 1:**
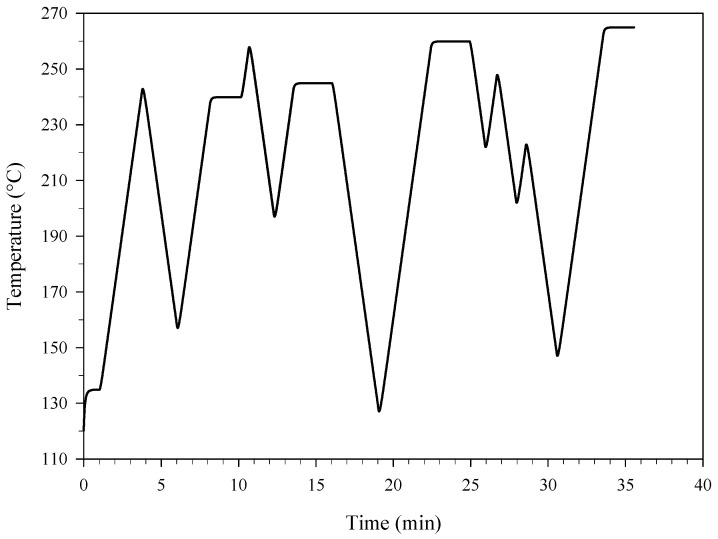
Temperature of PA as a function of time: non-isothermal accelerated aging test during which the sample was subjected to a complex thermal history.

The ability of the kinetics to predict the effect of thermal aging for PA was assessed against the conversion of artificially aged material, i.e., PA subjected to isothermal and non-isothermal accelerated aging tests, and naturally aged material, i.e., PA stored as dry powder at room temperature (in the Calorimetry Laboratory of CNR-STEMS) for more than 10 years. As detailed in Table 2, isothermal accelerated aging tests were performed over wide ranges of temperature and exposure time, by using the calorimeter used for the DSC experiments and a dry bath heater (THERMOBLOCK TD 200 P2+ by FALC Instruments, Treviglio, Italy). Specifically, the latter was used for tests carried out at lower temperatures for which the exposure times were much longer. Even in these tests, PA was loaded into the stainless steel pans used for DSC. A non-isothermal accelerated aging test was also performed by subjecting a sample of PA to the complex thermal history of Figure 1 (in the differential scanning calorimeter). Real life storage conditions often include a complex history of temperature variations. The reliability of the kinetic predictions was therefore assessed not only for isothermal conditions but also for time-varying temperature conditions during the aging treatment of PA.

**Table 2:** Conditions of isothermal accelerated aging tests.

Differential Scanning Calorimeter	Temperature (°C)	Exposure Time (min)
	200–230	25–180
Dry Bath Heater	Temperature (°C)	Exposure Time
	140	1 week and 5 weeks
	90	6 months

At the end of each test, the pan was recovered and the residue was dissolved in acetonitrile after quenching. In order to evaluate the conversion of substrate, the resulting solution was then subjected to high performance liquid chromatography (HPLC) analysis, which was performed using an Agilent 1100 device (Waldbronn, Germany) equipped with a binary pump and a Synergi 4 µm Hydro-RP 80 Å column by Phenomenex Inc. The elution conditions were as follows: a temperature of 30 °C; 30% (*v*/*v*) acetonitrile in an aqueous buffer solution with a pH of 2.5; and a flow rate of 1.0 mL min^−1^. The signal of PA was acquired at 210 nm and the retention time was 10.35 min under the elution conditions adopted. Naturally aged PA was also analyzed by HPLC after dissolution in acetonitrile.

## 3. Results and Discussion

### 3.1. Estimation and Validation of the Kinetics of Thermal Decomposition of Picric Acid (PA)

The rate of reaction for many thermally stimulated processes can be written as follows:

(1) $$\mathrm{rate}\;\mathrm{of}\;\mathrm{reaction}=\frac{d\alpha }{\mathrm{dt}}=k\left(T\right)\;f\left(\alpha \right)$$

where α is the conversion of substrate, t is time, k(T) is the reaction rate constant which is dependent on the absolute temperature, T, and f(α) is the kinetic law which describes the dependence of the rate of reaction on α. The relationship between k and T can be described with the Arrhenius equation:

(2) $$k=k\left(T\right)={A\;e}^{-\left(\frac{E_{a}}{\mathrm{RT}}\right)}$$

where A is the pre-exponential factor, $E_{a}$ is the activation energy, and R is the ideal gas constant. A model-based approach was adopted in this study; thus, f(α) was predetermined based on the thermal behavior of picric acid (PA). Estimating the kinetics of thermal decomposition in the frame of a model-based approach means quantifying A, $E_{a}$, and the parameters appearing in f(α).

**Figure 2:**
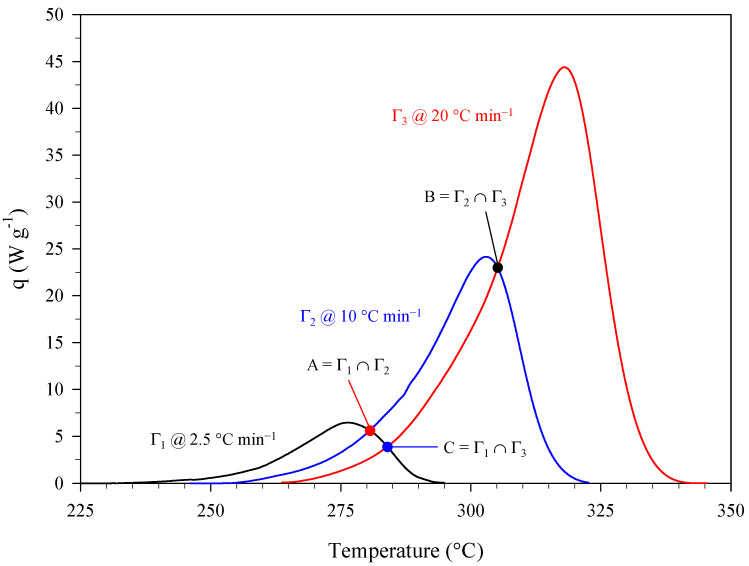
Specific heat power curves (mean curves) collected during a set of dynamic DSC experiments carried out on samples of PA at different heating rates.

Figure 2 shows the results of a set of dynamic differential scanning calorimetry (DSC) experiments carried out on PA at heating rates of 2.5, 10, and 20 °C min^−1^ in terms of curves “specific (i.e., referring to the mass of sample) heat power, q, versus temperature” (mean curves). The presence of intersection points (A, B, and C) between two curves obtained at different heating rates reveals the autocatalytic nature of the thermal decomposition of PA [15,16], confirming previous findings (see, e.g., [13]).

**Figure 3:**
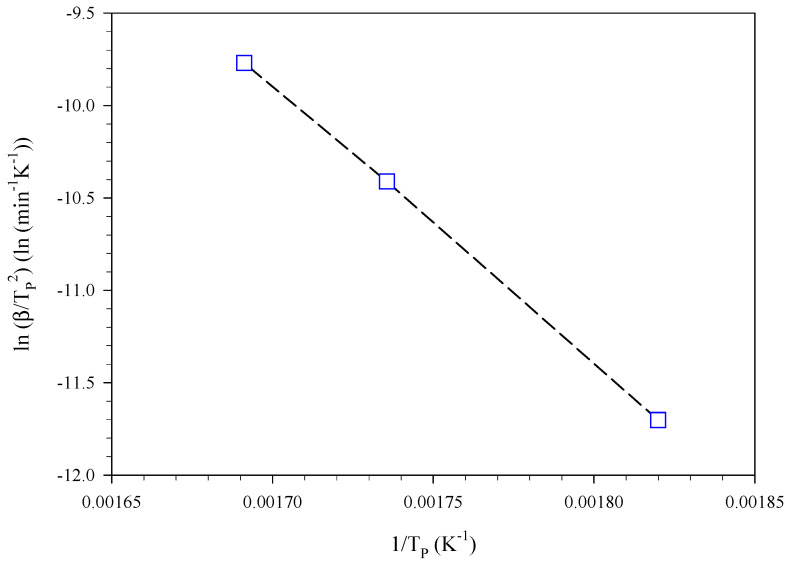
Kissinger plot corresponding to the curves of Figure 2.

A first estimate of the kinetic parameters, i.e., the pre-exponential factor, A, and activation energy, $E_{a}$, was obtained by applying the extended Kissinger method [17] to the curves of Figure 2. Figure 3 shows the resulting Kissinger plot. $T_{P}$ is the temperature that corresponds to the maximum of the specific heat power curve collected at a given heating rate, β. Based on the intercept and slope of this interpolation line, the values of A and $E_{a}$ were estimated to be equal to 1.98 × 10^11^ min^−1^ (i.e., 3.30 × 10^9^ s^−1^) and 1.25 × 10^6^ J mol^−1^, respectively. Furthermore, a value of reaction heat, ${\Delta H}_{R}$, equal to −3338 ± 145 J g^−1^ was obtained from the integration of the peaks of Figure 2.

**Figure 4:**
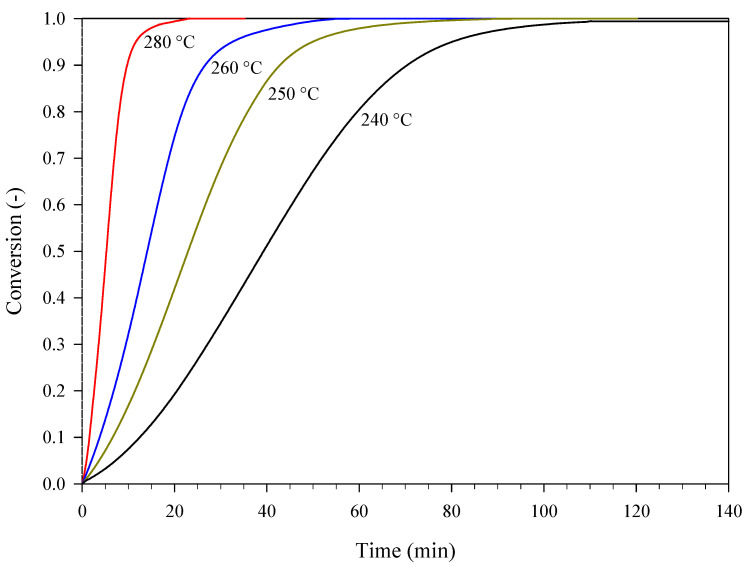
Conversion curves corresponding to specific heat power curves (mean curves) collected during a set of isothermal DSC experiments carried out on samples of PA at different temperatures.

PA was also subjected to isothermal DSC experiments at 240 °C, 250 °C, 260 °C, and 280 °C. In this case, the value of ${\Delta H}_{R}$ estimated from the mean specific heat power curves was found to be equal to −3483 ± 391 J g^−1^. This value is close to the value obtained in dynamic conditions. Figure 4 shows the conversion curves at different temperatures. The conversion can be calculated according to:

(3) $$\alpha =\frac{\int_{0}^{t}q\left(t\right)\mathrm{dt}}{\int_{0}^{\infty }q\left(t\right)\mathrm{dt}}=\frac{\int_{T_{0}}^{T}q\left(T\right)\mathrm{dT}}{\int_{T_{0}}^{T_{\infty }}q\left(T\right)\mathrm{dT}}$$

where q(t) is the specific heat power as a function of time (isothermal and dynamic conditions) and q(T) is the specific heat power as a function of temperature (dynamic conditions).

The autocatalytic nature of the process under examination is further confirmed by the sigmoidal shape of these conversion curves [15,16]. Consistently with this nature, the following expression of f(α) was assumed in Equation (1) [16]:

(4) $$f\left(\alpha \right)=\left(z+\alpha^{m}\right){\left(1-\alpha \right)}^{n}$$

where z is the autocatalytic factor and m and n are the reaction orders. Equation (4) represents a model of generalized autocatalysis [16,18]. The dynamic DSC data shown in Figure 2 (mean data) were used to estimate the kinetic triplet, i.e., A, $E_{a}$, and the parameters of the kinetic law, z, m, and n. The specific heat power, q, can be expressed as follows:

(5) $$q=\frac{d\alpha }{\mathrm{dt}}\;\left(-\Delta H_{R}\right)=K\left(T\right)\;f\left(\alpha \right)\;\left(-\Delta H_{R}\right)$$

The values of q given by Equation (5) were used to construct the sum of squared errors whose minimization with respect to the kinetic parameters provided their final estimate.

Assuming z=0 in Equation (4) and plotting (for the different combinations of i and j indices referring to two different heating rates) ${\;y}_{i,j}=\frac{\ln\left(\frac{q_{i}}{q_{j}}\right)}{\ln\left(\frac{\alpha_{i}}{\alpha_{j}}\right)}$ as a function of $x_{i,j}=\frac{\ln\left(\frac{1-\alpha_{i}}{1-\alpha_{j}}\right)}{\ln\left(\frac{\alpha_{i}}{\alpha_{j}}\right)}$ near the intersection points (A, B, and C) of the curves of Figure 2 leads to a first estimate of the reaction orders, m and n [15,16]. Table 3 gives these values along with the values of $E_{a}$ and A previously obtained using the extended Kissinger method.

Starting from the values of Table 3 and the data of Figure 2, a suitable identification procedure [16] was used to get the final estimate of the kinetic triplet, which is given in Table 4 in terms of mean values and 95% confidence intervals.

**Figure 5:**
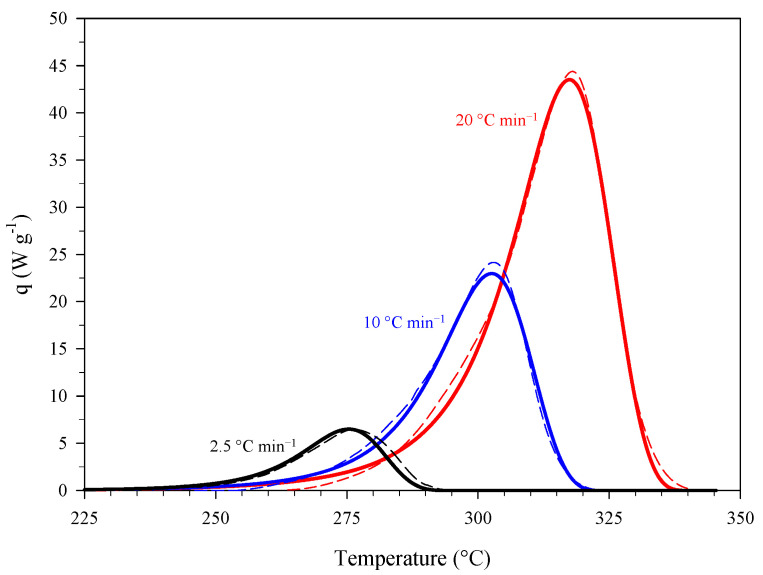
Specific heat power curves at different heating rates: DSC data of Figure 2 (mean data, dashed lines) and corresponding kinetic predictions (solid lines).

Figure 5 shows the comparison between experimental data at different heating rates (dashed lines) and corresponding kinetic predictions (solid lines). These predictions and those presented later in the manuscript were obtained by using the mean values of Table 4.

**Figure 6:**
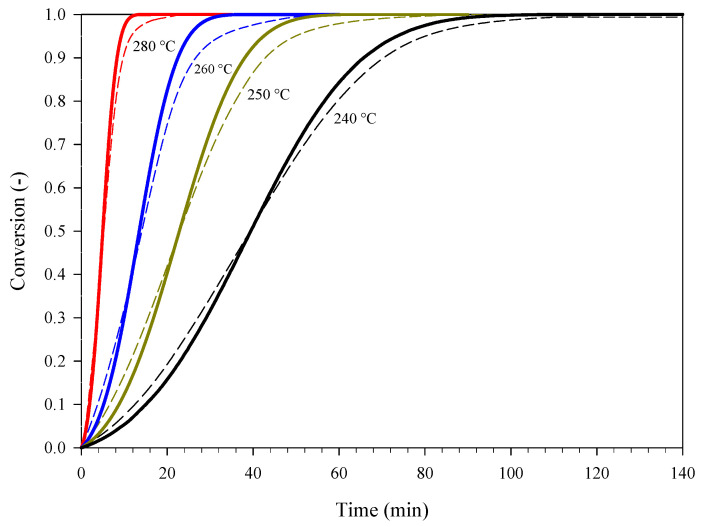
The conversion of PA as a function of time at different temperatures: DSC experiments (curves of Figure 4, dashed lines) and corresponding kinetic predictions (solid lines).

**Figure 7:**
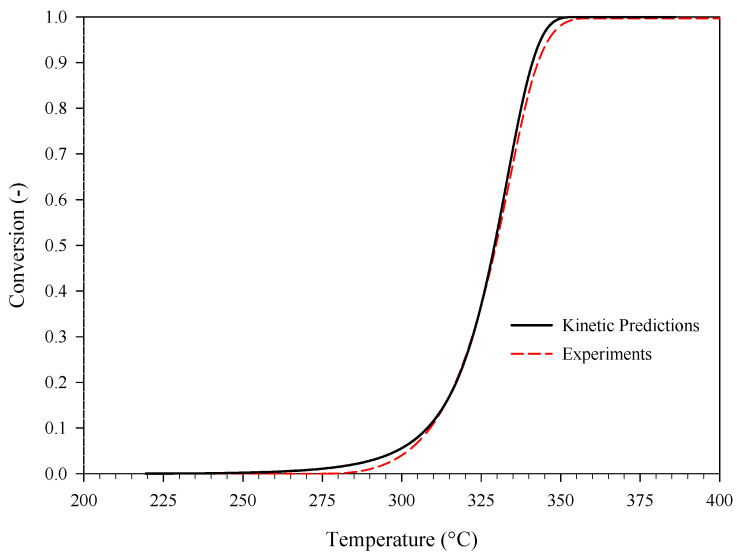
The conversion of PA as a function of temperature at a heating rate of 40 °C min^−1^: DSC experiments (mean data, dashed line) and corresponding kinetic predictions (solid line).

The obtained kinetics were validated against the conversion curve calculated from additional dynamic DSC data gathered at a heating rate of 40 °C min^−^**^1^** (mean data) and, more importantly, against the isothermal conversion curves of Figure 4. The comparison between experiments and corresponding kinetic predictions is shown in Figure 6 (isothermal conditions) and Figure 7 (dynamic conditions). Overall, these two figures show that the kinetic predictions are in good agreement with both the isothermal and the dynamic DSC experiments.

### 3.2. Kinetic Predictions for Aged PA

**Figure 8:**
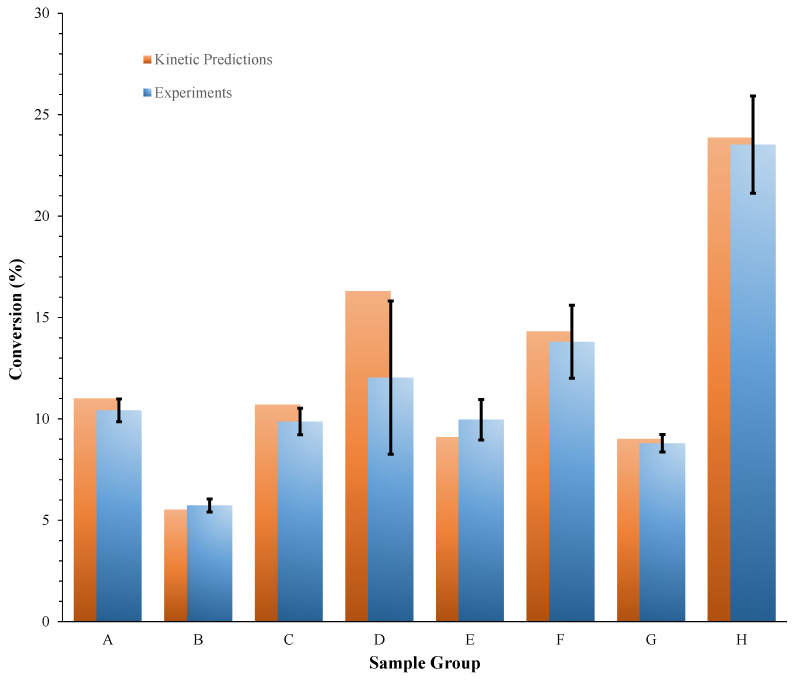
Conversions of PA evaluated using HPLC analysis for the different sample groups of Table 5 and the corresponding kinetic predictions.

The ability of the kinetics of thermal decomposition extracted to predict the conversion of aged PA was also assessed. Specifically, this assessment was first performed against the conversion of PA evaluated using high performance liquid chromatography (HPLC) analysis at the end of isothermal accelerated aging tests performed at the 200–230 °C temperature range. Aging tests were carried out in triplicate for each condition investigated. Table 5 details the values of conversion evaluated at the end of each test, $\alpha_{\mathrm{Test}}$, the mean conversion over three tests, $\alpha_{\mathrm{Experimental}}$, along with the standard deviation, σ, and conversion predicted by the kinetics, $\alpha_{\mathrm{Predicted}}$. The error, ε, is defined as follows:

(6) $$\varepsilon =\frac{\left(\alpha_{\mathrm{Experimental}}-\alpha_{\mathrm{Predicted}}\right)}{\alpha_{\mathrm{Predicted}}}\;100\;$$

and was also reported in Table 5. Figure 8 provides a more immediate representation of the comparison between experimental conversions for the different sample groups of Table 5 and the corresponding kinetic predictions.

**Table 5:** Conversions of PA evaluated using HPLC analysis at the end of isothermal accelerated aging tests performed at the 200–230 °C temperature range and corresponding kinetic predictions.

Sample Group	ID	m_PA_ (mg) *	T (°C)	Exposure Time (min)	$\alpha_{Test}$ (%)	$\alpha_{\mathrm{Experimental}}$ (%)	σ (%)	$\alpha_{\mathrm{Predicted}}$ (%)	ε (Equation (6)) (%)
A	1	5.5	200	180	10.8	10.4	0.6	11.0	−5.3
	2	2.1			10.7				
	3	4.3			9.8				
B	1	3.2	210	60	5.6	5.7	0.3	5.5	3.9
	2	4.0			5.5				
	3	4.2			6.1				
C	1	2.4	210	90	9.3	9.9	0.6	10.7	−7.7
	2	2.2			10.6				
	3	2.4			9.8				
D	1	2.3	210	120	14.7 14.0 14.9	14.5	0.5	16.3	−10.8
	2	2.1							
	3	2.2							
E	1	2.3	220	45	10.5	10.0	1.0	9.1	9.5
	2	6.2			8.8				
	3	3.2			10.6				
F	1	3.2	220	60	15.8	13.8	1.8	14.3	−3.6
	2	2.5			13.3				
	3	3.7			12.4				
G	1	4.9	230	25	9.2	8.8	0.4	9.0	−2.3
	2	3.1			8.4				
	3	2.8			8.8				
H	1	2.1	230	45	22.5	23.5	2.4	23.9	−1.4
	2	8.0			26.3				
	3	3.4			21.8				

* Mass of PA loaded into the pan.

The good agreement between predictions and experiments shown in Table 5 and Figure 8 is a first proof of the reliability of the kinetics adopted for simulating the effect of thermal aging for PA. This reliability was further proven. To this end, PA was kept at lower temperatures for longer time periods (1 week and 5 weeks at 140 °C and 6 months at 90 °C). Moreover, given that real life aging conditions may include temperature variations, PA was also subjected to the complex thermal history of Figure 1 (with temperatures varying between 120 °C and 265 °C over a time period of 35 min). In all cases, a good agreement was found between the kinetic predictions and the experimental data. Specifically, in the case of isothermal aging, the conversions evaluated using HPLC analysis were equal to 5.70% and 72.10% after one week and five weeks at 140 °C, respectively, whereas the corresponding predicted values were equal to 5.75% and 73.97%, respectively. In addition, in agreement with the experiments, a very low conversion of PA (~0.70%) was predicted by the kinetics after 6 months at 90 °C. In the case of non-isothermal aging, a conversion of PA equal to 35.87% was evaluated using HPLC analysis, whereas the corresponding predicted value was equal to 33.53%. These results highlight the versatility of the kinetics, which provided reliable predictions not only for isothermal conditions but also for time-varying temperature conditions during the aging treatment of PA.

The predictive ability of the kinetics of thermal decomposition from this study was also assessed against the conversion of naturally aged PA, i.e., PA aged at a much lower temperature and for a much longer time period than accelerated aging tests (i.e., room temperature for more than 10 years). This material was stored (as dry powder) in the Calorimetry Laboratory of CNR-STEMS. Its availability also justified the choice of PA as the energetic material to focus on in this study. The kinetics predicted a negligible conversion of substrate also confirmed by the HPLC analysis of this sample. This is consistent with the almost overlap of the DSC thermograms recorded under dynamic conditions for fresh (i.e., unaged) and naturally aged material [19].

**Figure 9:**
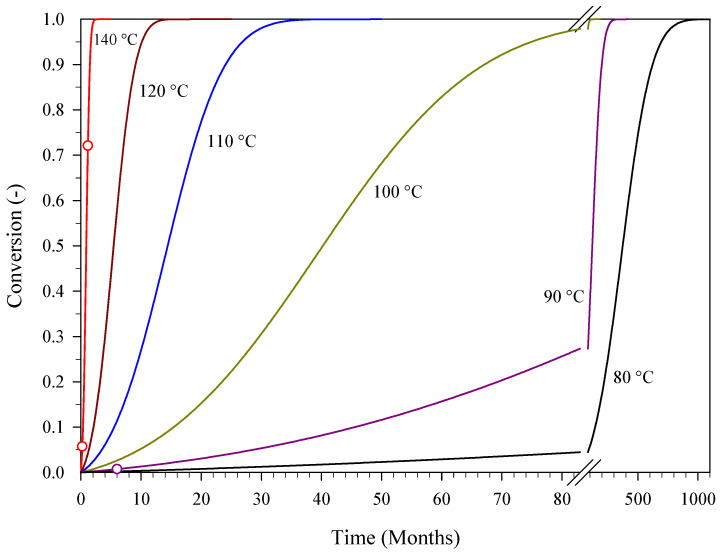
The conversion of PA versus time obtained via isothermal simulations performed with the kinetics from this study at different temperatures. Circles represent experimental data from accelerated aging tests.

The kinetics identified here allow for the prediction of shelf life once the failure threshold of PA has been defined as a function of the limiting extent of conversion. Figure 9 shows the conversion of substrate versus time as calculated via isothermal simulations performed at temperatures ranging from 80 °C to 140 °C. For example, when assuming 10% as the limiting extent of conversion, the shelf life of PA is about 4 months at 140 °C and about 12 years (143 months) at 80 °C.

Table 6 provides the kinetic predictions of the shelf life of PA at temperatures lower than 80 °C. These predictions confirm that the decomposition of PA (and, more generally, of aromatic nitro compounds) can occur at room temperature, but it is so slow that it becomes detectable only after thousands of years [1].

**Table 6:** Kinetic predictions of the shelf life of PA at different temperatures.

Temperature (°C)	70	60	50	40	30	20
Shelf Life (years) *	40	144	564	2411	11,287	58,800

* Calculated by assuming 10% as the limiting extent of conversion.

## 4. Conclusions

The thermal decomposition of picric acid (PA) was investigated in this study using differential scanning calorimetry (DSC) experiments which confirmed its autocatalytic nature. A kinetic law, Equation (4), consistent with this nature was assumed, and the kinetics of thermal decomposition were extracted via a suitable procedure of parameter identification from DSC data gathered under dynamic conditions at three different heating rates. In addition to satisfactorily reproducing isothermal and further dynamic DSC experiments, the obtained kinetics provided accurate predictions of the conversion of PA subjected to isothermal and non-isothermal accelerated aging tests (performed over wide ranges of temperatures and exposure times), which was evaluated using high performance liquid chromatography (HPLC) analysis. However, even more interestingly, it also predicted a negligible conversion for a sample of naturally aged PA, i.e., PA stored at room temperature for more than 10 years, which was confirmed by the HPLC analysis of this material.

In light of the results obtained, the methodology adopted in this study, which is based on the extraction of the kinetics of thermal decomposition from dynamic DSC data in the frame of a model-based approach incorporating Equation (4) as a kinetic law, is proposed to predict the shelf life of energetic materials that, like PA, decompose in an autocatalytic manner once their failure threshold has been defined as a function of the limiting extent of conversion. This is a reliable approach, as proven for PA, that can make it possible to avoid time- and money-consuming aging tests or, in any case, to minimize them through a reasoned planning of the experimental campaign.

## Figures and Tables

**Table 1:** Conditions of DSC experiments performed to estimate and validate the kinetics of the thermal decomposition of PA.

Estimation	Dynamic Conditions, Heating Rate (°C min^−1^)
	2.5; 10; 20
Validation	Isothermal Conditions, Temperature (°C)
	240; 250; 260; 280
	Dynamic Conditions, Heating Rate (°C min^−1^)
	40

**Table 3:** Kinetics of the thermal decomposition of PA: first estimate.

A (min^−1^)	$E_{a}\;(J\;{\mathrm{mol}}^{-1})$	m	n
1.98 × 10^11^	125,174	0.49	0.79

**Table 4:** Kinetics of the thermal decomposition of PA: final estimate.

	A (min^−1^)	$E_{a}\;(J\;{\mathrm{mol}}^{-1})$	z	m	n
Mean Value	1.52 × 10^11^	121,991	0.055	0.850	0.828
95% Confidence Interval	3.09 × 10^10^	1043	0.012	0.079	0.037

## Data Availability

The data presented in this study are available upon request from the authors.
